# Publisher Correction: Signatures of TOP1 transcription-associated mutagenesis in cancer and germline

**DOI:** 10.1038/s41586-022-04812-z

**Published:** 2022-05-03

**Authors:** Martin A. M. Reijns, David A. Parry, Thomas C. Williams, Ferran Nadeu, Rebecca L. Hindshaw, Diana O. Rios Szwed, Michael D. Nicholson, Paula Carroll, Shelagh Boyle, Romina Royo, Alex J. Cornish, Hang Xiang, Kate Ridout, The Genomics England Research Consortium, John C. Ambrose, Prabhu Arumugam, Roel Bevers, Marta Bleda, Freya Boardman-Pretty, Christopher R. Boustred, Helen Brittain, Mark J. Caulfield, Georgia C. Chan, Greg Elgar, Tom Fowler, Adam Giess, Angela Hamblin, Shirley Henderson, Tim J. P. Hubbard, Rob Jackson, Louise J. Jones, Dalia Kasperaviciute, Melis Kayikci, Athanasios Kousathanas, Lea Lahnstein, Sarah E. A. Leigh, Ivonne U. S. Leong, Javier F. Lopez, Fiona Maleady-Crowe, Meriel McEntagart, Federico Minneci, Loukas Moutsianas, Michael Mueller, Nirupa Murugaesu, Anna C. Need, Peter O’Donovan, Chris A. Odhams, Christine Patch, Mariana Buongermino Pereira, Daniel Perez-Gil, John Pullinger, Tahrima Rahim, Augusto Rendon, Tim Rogers, Kevin Savage, Kushmita Sawant, Richard H. Scott, Afshan Siddiq, Alexander Sieghart, Samuel C. Smith, Alona Sosinsky, Alexander Stuckey, Mélanie Tanguy, Ana Lisa Taylor Tavares, Ellen R. A. Thomas, Simon R. Thompson, Arianna Tucci, Matthew J. Welland, Eleanor Williams, Katarzyna Witkowska, Suzanne M. Wood, Colorectal Cancer Domain UK 100,000 Genomes Project, Daniel Chubb, Alex Cornish, Ben Kinnersley, Richard Houlston, David Wedge, Andreas Gruber, Anna Frangou, William Cross, Trevor Graham, Andrea Sottoriva, Gulio Caravagna, Nuria Lopez-Bigas, Claudia Arnedo-Pac, David Church, Richard Culliford, Steve Thorn, Phil Quirke, Henry Wood, Ian Tomlinson, Boris Noyvert, Anna Schuh, Konrad Aden, Claire Palles, Elias Campo, Tatjana Stankovic, Martin S. Taylor, Andrew P. Jackson

**Affiliations:** 1 Disease Mechanisms, MRC Human Genetics Unit, Institute of Genetics and Cancer, The University of Edinburgh, Edinburgh, UK; 2 Biomedical Genomics, MRC Human Genetics Unit, Institute of Genetics and Cancer, The University of Edinburgh, Edinburgh, UK; 3 Institut d’Investigacions Biomèdiques August Pi i Sunyer (IDIBAPS), Barcelona, Spain; 4 Centro de Investigación Biomédica en Red de Cáncer (CIBERONC), Madrid, Spain; 5 Institute of Cancer and Genomic Sciences, University of Birmingham, Birmingham, UK; 6 Cancer Research UK Edinburgh Centre, Institute of Genetics and Cancer, The University of Edinburgh, Edinburgh, UK; 7 Genome Regulation, MRC Human Genetics Unit, Institute of Genetics and Cancer, The University of Edinburgh, Edinburgh, UK; 8 Barcelona Supercomputing Center (BSC), Barcelona, Spain; 9 The Institute of Cancer Research, London, UK; 10 Institute of Clinical Molecular Biology, Christian-Albrechts-University and University Hospital Schleswig-Holstein, Kiel, Germany; 11 Department of Oncology, University of Oxford, Oxford, UK; 12 Hospital Clínic of Barcelona, Barcelona, Spain; 13 Departament de Fonaments Clínics, Universitat de Barcelona, Barcelona, Spain; 14 Genomics England, London, UK; 15 William Harvey Research Institute, Queen Mary University of London, London, UK; 16 Manchester Interdisciplinary Biocentre, University of Manchester, Manchester, UK; 17 Wellcome Centre for Human Genetics, Oxford, UK; 18 Cancer Institute, University College London, London, UK; 19 Barts Cancer Institute, Barts and The London School of Medicine and Dentistry, Queen Mary University of London, London, UK; 20 Institute for Research in Biomedicine (IRB Barcelona), The Barcelona Institute of Science and Technology (BIST), Barcelona, Spain; 21 Pathology and Data Analytics, Leeds Institute of Medical Research, St James’s University Hospital, University of Leeds, Leeds, UK

**Keywords:** Cancer genomics, Genomic instability, Nucleotide excision repair, Cancer genomics

Correction to: *Nature* 10.1038/s41586-022-04403-y Published online 9 February 2022

In the version of this article initially published, there were errors in the affiliations shown for Colorectal Cancer Domain UK 100,000 Genomes Project members Steve Thorn and Henry Wood. Steve Thorn is with the Cancer Research UK Edinburgh Centre, Institute of Genetics and Cancer, The University of Edinburgh, Edinburgh, UK (not University of Leeds). Henry Wood is with Pathology and Data Analytics, Leeds Institute of Medical Research, St James’s University Hospital, University of Leeds, Leeds, UK (not The University of Edinburgh). The errors have been corrected in the HTML and PDF versions of the article.

