# Supplementary material for: Signatures of TOP1 transcription-associated mutagenesis in cancer and germline
Source: Nature. 2022 Feb 9;602(7898):623–31. doi: 10.1038/s41586-022-04403-y (PMC8866115; doi:10.1038/s41586-022-04403-y)
Supplement: Supplementary file 1 — Supplementary Fig. 1, Tables 1–5 and references. [file 41586_2022_4403_MOESM1_ESM.pdf]

nature portfolio

https://doi.org/10.1038/s41586-022-04403-y

Supplementary information

Signatures of TOP1 transcription-associated mutagenesis in cancer and germline

In the format provided by the authors and unedited

Supplementary Information to:

Signatures of TOP1 transcription-associated mutagenesis in cancer and germline

Martin A.M. Reijns[1,14*], David A. Parry[1,14], Thomas C. Williams[1,2,14], Ferran Nadeu[3,4], Rebecca L. Hindshaw[5], Diana O. Rios Szwed[1], Michael D. Nicholson[6], Paula Carroll[1], Shelagh Boyle[7], Romina Royo[8], Alex J. Cornish[9], Hang Xiang[10], Kate Ridout[11], The Genomics England Research Consortium[+], Colorectal Cancer Domain UK 100,000 Genomes Project[+], Anna Schuh[11], Konrad Aden[10], Claire Palles[5], Elias Campo[3,4,12,13], Tatjana Stankovic[5], Martin S. Taylor[2*], Andrew P. Jackson[1*]

[1] Disease Mechanisms, MRC Human Genetics Unit, Institute of Genetics and Cancer, The University of Edinburgh, Edinburgh, UK

[2] Biomedical Genomics, MRC Human Genetics Unit, Institute of Genetics and Cancer, The University of Edinburgh, Edinburgh, UK

[3] Institut d'Investigacions Biomèdiques August Pi i Sunyer (IDIBAPS), Barcelona, Spain

[4] Centro de Investigación Biomédica en Red de Cáncer (CIBERONC), Madrid, Spain

[5] Institute of Cancer and Genomic Sciences, University of Birmingham, Edgbaston, UK

[6] Cancer Research UK Edinburgh Centre, Institute of Genetics and Cancer, The University of Edinburgh, Edinburgh, UK

[7] Genome Regulation, MRC Human Genetics Unit, Institute of Genetics and Cancer, The University of Edinburgh, Edinburgh, UK

[8] Barcelona Supercomputing Center (BSC), Barcelona, Spain

[9] The Institute of Cancer Research, London, UK

[10] Institute of Clinical Molecular Biology, Christian-Albrechts-University and University Hospital Schleswig-Holstein, Kiel, Germany

[11] Department of Oncology, University of Oxford, Oxford, UK

[12] Hospital Clínic of Barcelona, Barcelona, Spain

[13] Departament de Fonaments Clínics, Universitat de Barcelona, Barcelona, Spain

[14] These authors contributed equally: Martin A.M. Reijns, David A. Parry, Thomas C. Williams

[+] A list of authors and their affiliations appears at the end of the paper.

[*] Correspondence to MAMR, MST, APJ

Table of Contents

Supplementary Fig. 1 | Gel source data **page 3**

Supplementary Table 1 | Plasmids used in this study **page 4**

Supplementary Table 2 | *S. cerevisiae* strains used in this study **page 4**

Supplementary Table 3 | Oligonucleotides used in this study **page 5**

Supplementary Table 4 | Human cell lines used in this study **page 6**

Supplementary Table 5 | Published datasets used in this study **page 7,8**

Supplementary References **page 8,9**

Fig. 2b

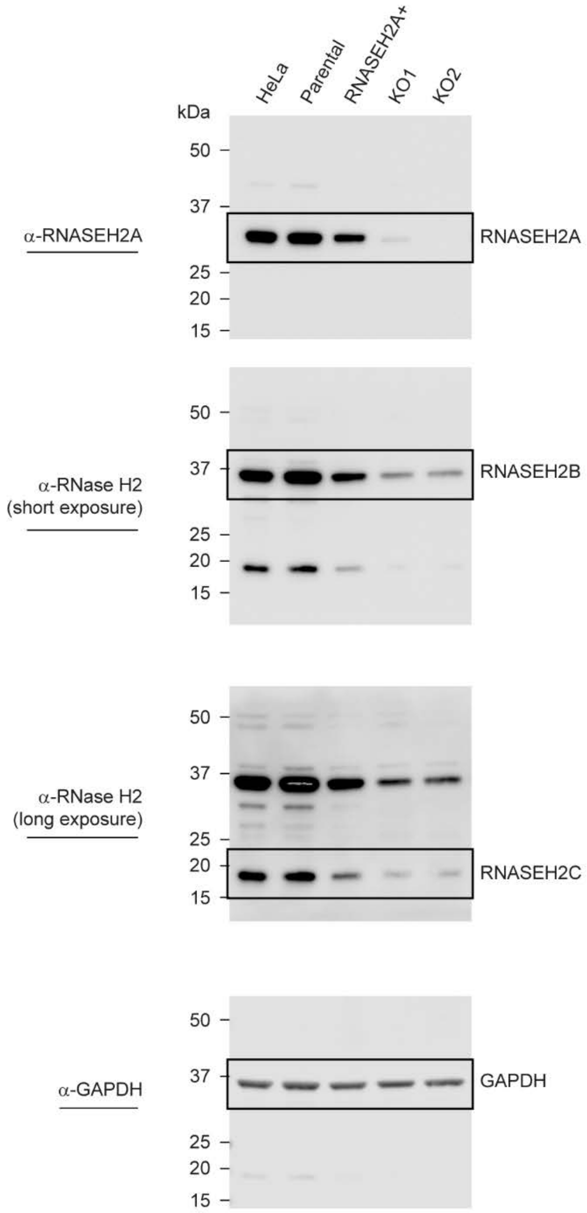

Extended Data Fig. 4a

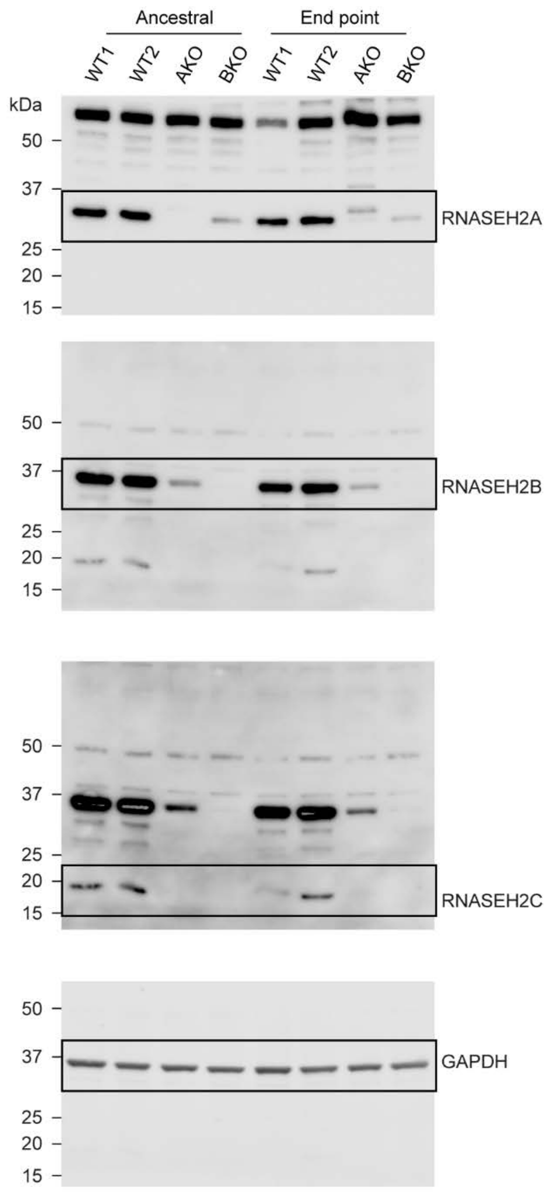

Supplementary Fig. 1 | Gel source data. For Fig. 2b and Extended Data Fig. 4a, samples were run on the same gel as the GAPDH loading control. Uncropped, full gel images are displayed in Extended Data Fig. 3a,b,f and Extended Data Fig. 4c, and contain labeled molecular weight markers; these are therefore not reproduced here.

Supplementary Table 1 | Plasmids[a] used in this study

Plasmid	Description	Reference
pFA6a-natMX6	For PCR-mediated gene disruption with nourseothricin resistance cassette	[1], a gift from Aziz El Hage
pFA6a-His3MX6	For PCR-mediated gene disruption with HIS3 nutritional selection cassette	[2], a gift from Jean Beggs
pTCW12	*S. cerevisiae* 2-bp deletion reporter, with SSTR-enriched HygroR (allows PCR-based amplification using MX6 primers for genomic integration)	This work (Fig. 1)
pTCW14	Gateway compatible Entry vector containing mammalian 2-bp deletion reporter, with SSTR-enriched HygroR	This work (Fig. 2)
pXAT2	Cas9 and AAVS1 sgRNA expression vector	[3], a gift from Knut Woltjen (Addgene plasmid # 80494)
pAAVS-Nst-CAG-Dest	Gateway Destination donor vector for AAVS1 targeting	[3], a gift from Knut Woltjen (Addgene plasmid # 80489)
pTCW15	Mammalian 2-bp deletion reporter from pTCW14 recombined into pAAVS-Nst-CAG-Dest (allows integration at the human AAVS1 locus when used in combination with pXAT2)	This work (Fig. 2, Extended Data Fig. 2 and 3)
pK18	A KanR plasmid, used for cloning and generating fluorescent in situ hybridisation (FISH) probes	[4], a gift from Laura Lettice
pTCW16	Human reporter construct (pTCW14/15) without the AAVS1 homology arms (*Hind*III/*Sca*I) cloned in pK18. Used for FISH probe synthesis	This work (Extended Data Fig. 3)
pX461	Contains Cas9n (D10A nickase mutant) from *S. pyogenes* with 2A-EGFP, and cloning backbone for sgRNA	[5], a gift from Feng Zhang (Addgene plasmid # 48140)
pX462	Contains Cas9n (D10A nickase mutant) from *S. pyogenes* with 2A-Puro, and cloning backbone for sgRNA	[5], a gift from Feng Zhang (Addgene plasmid # 48141)
pX461-RNASEH2A-gRNA1 (pMAR526)	Expresses guide RNA targeting exon 1 of human RNASEH2A	[6,7]
pX462-RNASEH2A-gRNA2 (pMAR527)	Expresses guide RNA targeting just downstream of human RNASEH2A exon 1	[6,7]

[a] Plasmids generated for this work available on request

Supplementary Table 2 | *S. cerevisiae* strains[a] used in this study

Name	Relevant features	Genotype
BY4741	Parental strain	MATa his3Δ1 leu2Δ0 met15Δ ura3Δ0
TCWY16	Wild type strain with 2-bp deletion reporter	BY4741, agp1::HpH-2bp-del-reporter(from pTCW12)
TCWY18	*top1Δ* strain with 2-bp deletion reporter	BY4741, top1Δ::natMX6 agp1::HpH-2bp-del-reporter(from pTCW12)
TCWY19	*rnh201Δ top1Δ* strain with 2-bp deletion reporter	BY4741, rnh201Δ::natMX6 top1Δ::His3MX6 agp1::HpH-2bp-del-reporter(from pTCW12)
TCWY20	*rnh201Δ* strain with 2-bp deletion reporter	BY4741 rnh201Δ::natMX6 agp1::HpH-2bp-del-reporter(from pTCW12)

[a] Yeast strains generated for this work available on request

Supplementary Table 3 | Oligonucleotides used in this study

Name	Sequence	Used for
AGP1-MX6-F	TGGGTTATTGGTCGGTAACGGTACCGC GTTGGTTCATGCGGGTCCAGCTGGACT ACTTATCGGATCCCCGGGTTAATTAAG	Amplification of 2-bp deletion reporter construct from pTCW12 for insertion at the *AGP1* locus
AGP1-MX6-R	CTTCTTGCTTGATTAATTCTTCATCAAA GATTTGTCTATGAGAATCTAGGTCGAT CTTGTGAATTCGAGCTCGTTTAAAC	Amplification of 2-bp deletion reporter construct from pTCW12 for insertion at the *AGP1* locus
S24F	ACGGATCCCCGGGTTAAT	Sequencing of yeast 2-bp deletion reporter
S297F	CACAGACGCGTTGAATTGTC	Sequencing of yeast 2-bp deletion reporter
S752F	CAAGATCTCCCAGAGACAGAGC	Sequencing of 2-bp deletion reporter
S1258F	TGGTCTCGACCAACTATACCAG	Sequencing of 2-bp deletion reporter
S1793F	AAAGGTAGCGTTGCCAATGA	Sequencing of yeast 2-bp deletion reporter
S2298F	ACCAGGATCTTGCCATCCTA	Sequencing of yeast 2-bp deletion reporter
S142R	GCACGTCAAGACTGTCAAGG	Sequencing of yeast 2-bp deletion reporter
S588R	CATATCCTCTCCCTCCCACA	Sequencing of 2-bp deletion reporter
S1113R	ACATCGCCTCTGACCAGTCT	Sequencing of 2-bp deletion reporter
S1658R	GCCTCGAAACGTGAGTCTTT	Sequencing of yeast 2-bp deletion reporter
S2136R	CCATTACGCTCGTCATCAAA	Sequencing of yeast 2-bp deletion reporter
S2652R	CGACAGCAGTATAGCGACCA	Sequencing of yeast 2-bp deletion reporter
dna803	TCGACTTCCCCTCTTCCGATG	Extended Data Fig. 3
dna804	GAGCCTAGGGCCGGGATTCTC	Extended Data Fig. 3
dna183	CTCAGGTTCTGGGAGAGGGTAG	Extended Data Fig. 3
Puro-F	GTCACCGAGCTGCAAGAACTC	Extended Data Fig. 3
Puro-3F	GCGCACCTGGTGCATGACC	Extended Data Fig. 3
HA-doR	GAGTTTGCCAAGCAGTCACC	Extended Data Fig. 3
HygroR_up	CAACGTGCTGGTTATTGTGC	Amplification and sequencing of human 2-bp deletion reporter
PuroR_rev	CAGCCTGCACCTGAGGAGTG	Amplification and sequencing of human 2-bp deletion reporter
H1443F	GTCACCGAGCTGCAAGAACT	Sequencing of human 2-bp deletion reporter
H1755F	GTCGAGGTGCCCGAAGGAC	Sequencing of human 2-bp deletion reporter
H1327R	GTGGGCTTGTACTCGGTCAT	Sequencing of human 2-bp deletion reporter
RNASEH2A-ex1F	ACCCGCTCCTGCAGTATTAG	Amplification and sequencing across *RNASEH2A* exon 1 to check for CRISPR/Cas9 genome editing
RNASEH2A-ex1R	TCCCTTGGTGCAGTGCAATC	Amplification and sequencing across *RNASEH2A* exon 1 to check for CRISPR/Cas9 genome editing

Supplementary Table 4 | Human cell lines[a] used in this study

Relevant features	Description	Reference
HeLa parental reporter cells	HeLa cell clone with mammalian 2-bp deletion reporter (from pTCW15) integrated at a single AAVS1 allele	This work
HeLa RNASEH2A-KO reporter clone 1	RNase H2 null clone. HeLa reporter cells with CRISPR/Cas9-mediated *RNASEH2A* knockout. Three loss of function mutations in exon 1 identified by capillary sequencing: 46 bp deletion, 39 bp deletion (deleting splice donor site), in-frame 21 bp deletion covering catalytic site residue D34)	This work
HeLa RNASEH2A-KO reporter clone 2	RNase H2 null clone. HeLa reporter cells with CRISPR/Cas9-mediated *RNASEH2A* knockout confirmed by capillary sequencing. Mutations in exon 1: 38 bp deletion, 14 bp deletion + 92 bp insertion.	This work
HeLa RNASEH2A+ reporter clone	RNase H2 proficient clone, resulting from CRISPR/Cas9 genome editing of HeLa parental reporter cells. Capillary sequencing detected in-frame 9 bp deletion in exon 1 not involving catalytic site residues + intronic 24 bp duplication mutation; 1 bp insertion + 11 bp deletion.	This work
HeLa RNASEH2A-KO control clone	RNase H2 null clone. Previously published CRISPR/Cas9-mediated *RNASEH2A* knockout clone	[6,7]
hTERT-RPE1 TP53-KO	hTERT-RPE1 (ATCC) with CRISPR/Cas9-mediated *TP53* knockout	[7], a gift from D. Durocher
hTERT-RPE1 TP53-KO RNASEH2A-KO	hTERT-RPE1 TP53-KO with CRISPR/Cas9-mediated *RNASEH2A* knockout (homozygous for 4 bp deletion in exon 3)	[7], a gift from D. Durocher
hTERT-RPE1 TP53-KO RNASEH2B-KO	hTERT-RPE1 TP53-KO with CRISPR/Cas9-mediated *RNASEH2B* knockout (homozygous for 1 bp insertion in exon 5)	[7], a gift from D. Durocher

[a] Cell lines generated for this work available on request

Supplementary Table 5 | Published datasets used in this study

Description	Origin	Reference
WGS for MLH-KO organoids (Extended Data Fig. 1)	VCF files shared by R. van Boxtel, University Medical Center Utrecht, The Netherlands	8
Categorised indels for WGS of ovarian adenocarcinoma from ICGC/TCGA PCAWG consortium somatic mutation calls (Extended Data Fig. 1)	https://dcc.icgc.org/releases/PCAWG/mutational_signatures/Input_Data_PCAWG7_23K_Spectra_DB/Mutation_Catalogs_--_Spectra_of_Individual_Tumours	9
WGS for *S. cerevisiae rnh201Δ pol2-M644G* (Fig. 1, Extended Data Fig. 7)	https://www.ncbi.nlm.nih.gov/sra/?term=SRP062900 (NCBI SRA database, study no. SRP062900)	10
delta|(-2)|-7B-YUNI300 *S. cerevisiae* reference genome	https://www.ncbi.nlm.nih.gov/geo/query/acc.cgi?acc=GSE56939 (NCBI GEO, accession number GSE56939)	11
WGS for CLL and irinotecan treated and untreated CRC (Genomics England 100,000 Genomes Project)[a] (Fig. 5, Extended Data Fig. 8)	Primary data from the 100,000 Genomes Project, which are held in a secure Research Environment, are available to registered users. For further information, see https://www.genomicsengland.co.uk/about-gecip/for-gecip-members/data-and-data-access/	12
WGS for CLL (ICGC) (Fig. 5, Extended Data Fig. 8)	https://ega-archive.org/studies/EGAS00001001306 (European Genome-Phenome Archive, accession number EGAS00001001306)	13
PCAWG indels (Fig. 5, Extended Data Fig. 8)	https://dcc.icgc.org/api/v1/download?fn=/PCAWG/consensus_snv_indel/final_consensus_passonly.snv_mnv_indel.icgc.public.maf.gz	14
PCAWG RNA-seq mRNA baseline (Fig. 5, Extended Data Fig. 8)	ArrayExpress E-MTAB-5200 https://www.ebi.ac.uk/gxa/experiments-content/E-MTAB-5200/resources/ExperimentDownloadSupplier.RnaSeqBaseline/tpms.tsv; downloaded on 27th July 2021	9
Human de novo mutations from Gene4Denovo (Fig. 5, Extended Data Fig. 9)	http://www.genemed.tech/gene4denovo/uploads/All_De_novo_mutations_1.2.txt	15
Human germ cell transcriptome data (Fig. 5)	https://www.ncbi.nlm.nih.gov/geo/query/acc.cgi?acc=GSE125372 (NCBI GEO database, accession code GSE125372)	16
TOP1-seq (Fig. 5, Extended Data Fig. 8)	https://www.ncbi.nlm.nih.gov/geo/query/acc.cgi?acc=GSE57628 (NCBI GEO database, accession code GSE57628; samples GSM1385717 and GSM1385718)	17
emRiboSeq *rnh201Δ* yeast (Extended Data Fig. 7)	https://www.ncbi.nlm.nih.gov/geo/query/acc.cgi?acc=GSE64521 (Sequence Read Archive sample accession codes: SRX824147, SRX824139, SRX824136, SRX824134)	18
Human GRCh37 reference genome	ftp://ftp-trace.ncbi.nih.gov/1000genomes/ftp/technical/reference/phase2_reference_assembly_sequence/hs37d5.fa.gz	
Human hg38 reference genome	ftp://hgdownload.cse.ucsc.edu/goldenPath/hg38/bigZips/hg38.fa.gz	
Mouse GRCm38 reference genome	ftp://ftp-mouse.sanger.ac.uk/ref/GRCm38_68.fa.gz	19
Mouse Indel and Structural Variant Data	ftp://ftp-mouse.sanger.ac.uk/REL-1505-SNPs_Indels/mgp.v5.merged.indels.dbSNP142.normed.vcf.gz and ftp://ftp-mouse.sanger.ac.uk/REL-1606-SV/mgpv5.SV_insertions.bed.gz and ftp://ftp-mouse.sanger.ac.uk/REL-1606-SV/mgpv5.SV_deletions.bed.gz	19
Human Short Polymorphism Data	https://ftp.ncbi.nih.gov/snp/organisms/human_9606_b151_GRCh37p13/VCF/All_20180423.vcf.gz	20
Human Structural Variant Data	https://hgdownload.soe.ucsc.edu/gbdb/hg38/bbi/dbVar	21
Human gene annotations	Ensembl (https://www.ensembl.org, ftp://ftp.ensembl.org/pub/release-90/gtf/homo_sapiens/Homo_sapiens.GRCh38.90.gtf.gz	22,23

	and http://ftp.ensembl.org/pub/release-75/gtf/homo_sapiens/Homo_sapiens.GRCh37.75.gtf.gz) and GENCODE (https://ftp.ebi.ac.uk/pub/databases/gencode/Gencode_human/release_38/gencode.v38.annotation.gff3.gz)	
Mouse gene annotations	GENCODE https://ftp.ebi.ac.uk/pub/databases/gencode/Gencode_mouse/release_M25/gencode.vM25.annotation.gff3.gz	24
Genome mappability data	https://bismap.hoffmanlab.org	25

[a] CLL and CRC data from the 100,000 Genomes Project are held in a secure Research Environment to protect participant privacy and can be accessed by joining an appropriate GECIP Domain using the application form at https://www.genomicsengland.co.uk/join-a-gecip-domain/. Detailed information on accessing 100,000 Genomes Project data including expected application timeframes and data use restrictions can be found at https://research-help.genomicsengland.co.uk/display/OC/GeCIP+and+your+access+to+data.

Supplementary References

1 Hentges, P., Van Driessche, B., Tafforeau, L., Vandenhaute, J. & Carr, A. M. Three novel antibiotic marker cassettes for gene disruption and marker switching in Schizosaccharomyces pombe. *Yeast* **22**, 1013-1019, doi:10.1002/yea.1291 (2005).

2 Wach, A., Brachat, A., Alberti-Segui, C., Rebischung, C. & Philippsen, P. Heterologous HIS3 marker and GFP reporter modules for PCR-targeting in Saccharomyces cerevisiae. *Yeast* **13**, 1065-1075, doi:10.1002/(SICI)1097-0061(19970915)13:11<1065::AID-YEA159>3.0.CO;2-K (1997).

3 Oceguera-Yanez, F. *et al.* Engineering the AAVS1 locus for consistent and scalable transgene expression in human iPSCs and their differentiated derivatives. *Methods* **101**, 43-55, doi:10.1016/j.ymeth.2015.12.012 (2016).

4 Pridmore, R. D. New and versatile cloning vectors with kanamycin-resistance marker. *Gene* **56**, 309-312, doi:10.1016/0378-1119(87)90149-1 (1987).

5 Ran, F. A. *et al.* Genome engineering using the CRISPR-Cas9 system. *Nature Protocols* **8**, 2281-2308, doi:10.1038/nprot.2013.143 (2013).

6 Benitez-Guijarro, M. *et al.* RNase H2, mutated in Aicardi-Goutières syndrome, promotes LINE-1 retrotransposition. *The EMBO journal* **37**, doi:10.15252/embj.201798506 (2018).

7 Zimmermann, M. *et al.* CRISPR screens identify genomic ribonucleotides as a source of PARP-trapping lesions. *Nature* **559**, 285-289, doi:10.1038/s41586-018-0291-z (2018).

8 Drost, J. *et al.* Use of CRISPR-modified human stem cell organoids to study the origin of mutational signatures in cancer. *Science* **358**, 234-238, doi:10.1126/science.aao3130 (2017).

9 Consortium, T. I. T. P.-C. A. o. W. G. Pan-cancer analysis of whole genomes. *Nature* **578**, 82-93, doi:10.1038/s41586-020-1969-6 (2020).

10 Conover, H. N. *et al.* Stimulation of Chromosomal Rearrangements by Ribonucleotides. *Genetics* **201**, 951-961, doi:10.1534/genetics.115.181149 (2015).

11 Lujan, S. A. *et al.* Heterogeneous polymerase fidelity and mismatch repair bias genome variation and composition. *Genome research* **24**, 1751-1764, doi:10.1101/gr.178335.114 (2014).

12 Consortium, T. G. E. R. The 100,000 Genomes Project | Genomics England. (2020).

13 Puente, X. S. *et al.* Non-coding recurrent mutations in chronic lymphocytic leukaemia. *Nature* **526**, 519-524, doi:10.1038/nature14666 (2015).

14 Alexandrov, L. B. *et al.* The repertoire of mutational signatures in human cancer. *Nature* **578**, 94-101, doi:10.1038/s41586-020-1943-3 (2020).

15 Zhao, G. *et al.* Gene4Denovo: an integrated database and analytic platform for de novo mutations in humans. *Nucleic acids research* **48**, D913--D926 (2020).

16 Xia, B. *et al.* Widespread Transcriptional Scanning in the Testis Modulates Gene Evolution Rates. *Cell* **180**, 248-262 e221, doi:10.1016/j.cell.2019.12.015 (2020).
17 Baranello, L. *et al.* RNA Polymerase II Regulates Topoisomerase 1 Activity to Favor Efficient Transcription. *Cell* **165**, 357-371, doi:10.1016/j.cell.2016.02.036 (2016).
18 Reijns, M. A. M. *et al.* Lagging-strand replication shapes the mutational landscape of the genome. *Nature* **518**, 502-506, doi:10.1038/nature14183 (2015).
19 Keane, T. M. *et al.* Mouse genomic variation and its effect on phenotypes and gene regulation. *Nature* **477**, 289-294, doi:10.1038/nature10413 (2011).
20 Sherry, S. T. *et al.* dbSNP: the NCBI database of genetic variation. *Nucleic Acids Res* **29**, 308-311, doi:10.1093/nar/29.1.308 (2001).
21 Lappalainen, I. *et al.* DbVar and DGVa: public archives for genomic structural variation. *Nucleic Acids Res* **41**, D936-941, doi:10.1093/nar/gks1213 (2013).
22 Aken, B. L. *et al.* Ensembl 2017. *Nucleic Acids Res* **45**, D635-D642, doi:10.1093/nar/gkw1104 (2017).
23 Flicek, P. *et al.* Ensembl 2014. *Nucleic Acids Res* **42**, D749-755, doi:10.1093/nar/gkt1196 (2014).
24 Frankish, A. *et al.* GENCODE reference annotation for the human and mouse genomes. *Nucleic Acids Res* **47**, D766-D773, doi:10.1093/nar/gky955 (2019).
25 Karimzadeh, M., Ernst, C., Kundaje, A. & Hoffman, M. M. Umap and Bismap: quantifying genome and methylome mappability. *Nucleic Acids Res* **46**, e120, doi:10.1093/nar/gky677 (2018).
