# Supplementary material for: Signatures of TOP1 transcription-associated mutagenesis in cancer and germline
Source: Nature. 2022 Feb 9;602(7898):623–31. doi: 10.1038/s41586-022-04403-y (PMC8866115; doi:10.1038/s41586-022-04403-y)
Supplement: Supplementary file 2 — Reporting Summary [file 41586_2022_4403_MOESM2_ESM.pdf]

nature portfolio

Corresponding author(s): Martin A.M. Reijns
Martin S. Taylor
Andrew P. Jackson

Last updated by author(s): Dec 9, 2021

# Reporting Summary

Nature Portfolio wishes to improve the reproducibility of the work that we publish. This form provides structure for consistency and transparency in reporting. For further information on Nature Portfolio policies, see our Editorial Policies and the Editorial Policy Checklist.

## Statistics

For all statistical analyses, confirm that the following items are present in the figure legend, table legend, main text, or Methods section.

| n/a | Confirmed | |
|---|---|---|
| ☐ | ☒ | The exact sample size (*n*) for each experimental group/condition, given as a discrete number and unit of measurement |
| ☐ | ☒ | A statement on whether measurements were taken from distinct samples or whether the same sample was measured repeatedly |
| ☐ | ☒ | The statistical test(s) used AND whether they are one- or two-sided<br>*Only common tests should be described solely by name; describe more complex techniques in the Methods section.* |
| ☒ | ☐ | A description of all covariates tested |
| ☐ | ☒ | A description of any assumptions or corrections, such as tests of normality and adjustment for multiple comparisons |
| ☐ | ☒ | A full description of the statistical parameters including central tendency (e.g. means) or other basic estimates (e.g. regression coefficient) AND variation (e.g. standard deviation) or associated estimates of uncertainty (e.g. confidence intervals) |
| ☐ | ☒ | For null hypothesis testing, the test statistic (e.g. *F*, *t*, *r*) with confidence intervals, effect sizes, degrees of freedom and *P* value noted<br>*Give P values as exact values whenever suitable.* |
| ☒ | ☐ | For Bayesian analysis, information on the choice of priors and Markov chain Monte Carlo settings |
| ☒ | ☐ | For hierarchical and complex designs, identification of the appropriate level for tests and full reporting of outcomes |
| ☐ | ☒ | Estimates of effect sizes (e.g. Cohen's *d*, Pearson's *r*), indicating how they were calculated |

*Our web collection on statistics for biologists contains articles on many of the points above.*

## Software and code

Policy information about availability of computer code

| | |
|---|---|
| Data collection | No software was used for data collection |
| Data analysis | Sequencher v5.4.6; Mutation Surveyor v3.30; AIDA Image Analyzer v3.44.035; GraphPad Prism v9.1.1; Microsoft Excel 2016; Python v3.8.5; R v3.3.1/v4.0.5/v4.1.0; SRA Toolkit v2.5.4-1; BWA-MEM v0.7.12/v0.7.15/v0.7.16; SAMBLASTER v0.1.22; GATK v3.6-0/v4.0.2.0/v4.1.9.0; Somalier v0.2.12; ISAAC aligner v03.16.02.19; cgpBattenberg v3.2.2;ascatNgs v4.1.0; Nexus 9.0 Biodiscovery; Strelka v2.1.9.10/v2.4.7/v2.8.2; SvABA v1.1.0/v1.1.3; Platypus v0.8.1; Manta v0.28.0/v1.2/v1.6.0; Canvas v1.3.1; biobambam2 v2.0.65; IGV v2.5.0; bcftools v1.10.2; Bedtools v2.30.0; samtools v1.9; liftover (kent source version 417); Logomaker v0.8; SigProfilerExtractor v1.1.0; SigprofilerMatrixGenerator v1.1.14/1.1.15; SigprofilerPlotting v1.1.27; scipy v1.6.3. Custom code available at https://git.ecdf.ed.ac.uk/ID-TOP1 |

For manuscripts utilizing custom algorithms or software that are central to the research but not yet described in published literature, software must be made available to editors and reviewers. We strongly encourage code deposition in a community repository (e.g. GitHub). See the Nature Portfolio guidelines for submitting code & software for further information.

## Data

Policy information about availability of data

All manuscripts must include a data availability statement. This statement should provide the following information, where applicable:
- Accession codes, unique identifiers, or web links for publicly available datasets
- A description of any restrictions on data availability
- For clinical datasets or third party data, please ensure that the statement adheres to our policy

RPE1 mutation accumulation experiment and mouse tumour WGS data are available from European Nucleotide Archive under accession number PRJEB48753 (https://www.ebi.ac.uk/ena/browser/view/PRJEB48753).

CLL and CRC data from the 100,000 Genomes Project are held in a secure Research Environment to protect participant privacy and can be accessed by joining an appropriate GECIP Domain using the application form at https://www.genomicsengland.co.uk/join-a-gecip-domain/. Detailed information on accessing 100,000 Genomes Project data including expected application timeframes and data use restrictions can be found at https://research-help.genomicsengland.co.uk/display/OC/GeCIP+and+your+access+to+data.
ICGC-CLL data is available at the European Genome-Phenome Archive (EGA, http://www.ebi.ac.uk/ega/), hosted at the EBI, under accession number EGAS00001001306.
ICGC PCAWG somatic mutations and mutational signature data were obtained from https://dcc.icgc.org/releases/PCAWG.
ICGC PCAWG "baseline" gene expression data were obtained from ArrayExpress (https://www.ebi.ac.uk/arrayexpress/experiments/E-MTAB-5200/)
Genome sequencing data from S. cerevisiae rnh201Δ pol2-M644G strains were obtained from NCBI SRA database, study no. SRP062900 (https://www.ncbi.nlm.nih.gov/sra?linkname=bioproject_sra_all&from_uid=293995).
Human de novo mutations were downloaded from the Gene4Denovo database (http://genemed.tech/gene4denovo/download).
Human germ cell transcriptome data is available at the NCBI GEO database (accession code GSE125372) and as a supplementary table from https://www.cell.com (https://www.cell.com/cms/10.1016/j.cell.2019.12.015/attachment/08d8d7db-2f52-499b-999e-4be5d6316e71/mmc5.xlsx).
Top1-seq data were obtained from NCBI GEO database, accession code GSE57628; samples GSM1385717 and GSM1385718 (https://www.ncbi.nlm.nih.gov/geo/query/acc.cgi?acc=GSE57628).
emRiboSeq data from rnh201Δ yeast were obtained from NCBI SRA database (https://www.ncbi.nlm.nih.gov/sra), accession codes SRX824147, SRX824139, SRX824136 and SRX824134.
Human GRCh37 reference genome sequence was obtained from ftp://ftp-trace.ncbi.nih.gov/1000genomes/ftp/technical/reference/phase2_reference_assembly_sequence/hs37d5.fa.gz.
Human hg38 reference genome was obtained from ftp://hgdownload.cse.ucsc.edu/goldenPath/ hg38/bigZips/hg38.fa.gz.
Mouse GRCm38 reference genome was obtained from ftp://ftp-mouse.sanger.ac.uk/ref/GRCm38_68.fa.gz.
The delta|(-2)|-7B-YUNI300 S. cerevisiae reference genome was obtained from NCBI GEO accession GSE56939 (https://ftp.ncbi.nlm.nih.gov/geo/series/GSE56nnn/GSE56939/suppl/GSE56939_L03_ref_v2.fa.gz).
Gene annotations were obtained from Ensembl (https://www.ensembl.org, ftp://ftp.ensembl.org/pub/release-90/gtf/homo_sapiens/Homo_sapiens.GRCh38.90.gtf.gz and http://ftp.ensembl.org/pub/release-75/gtf/homo_sapiens/Homo_sapiens.GRCh37.75.gtf.gz) and from GENCODE (https://ftp.ebi.ac.uk/pub/databases/gencode/Gencode_human/release_38/gencode.v38.annotation.gff3.gz and https://ftp.ebi.ac.uk/pub/databases/gencode/Gencode_mouse/release_M25/gencode.vM25.annotation.gff3.gz).
Mouse short indel and structural variant data were obtained from the Mouse Genomes Project (https://ftp.ncbi.nih.gov/snp/organisms/human_9606_b151_GRCh37p13/VCF/All_20180423.vcf.gz).
Human short polymorphism data were obtained from dbSNP151 (https://ftp.ncbi.nih.gov/snp/organisms/human_9606_b151_GRCh37p13/VCF/All_20180423.vcf.gz).
Human Structural Variant Data were obtained from dbVar (https://hgdownload.soe.ucsc.edu/gbdb/hg38/bbi/dbVar).
Genome mappability data was downloaded from https://bismap.hoffmanlab.org.

# Field-specific reporting

Please select the one below that is the best fit for your research. If you are not sure, read the appropriate sections before making your selection.

☒ Life sciences ☐ Behavioural & social sciences ☐ Ecological, evolutionary & environmental sciences

For a reference copy of the document with all sections, see nature.com/documents/nr-reporting-summary-flat.pdf

# Life sciences study design

All studies must disclose on these points even when the disclosure is negative.

| | |
|---|---|
| Sample size | No statistical methods were used to pre-determine sample size. Sample sizes for fluctuation assays were performed in line with standard practice in the field (doi.org/10.1385/1-59259-761-0:003). Samples sizes for the mutation accumulation experiment were determined based on the practicality of large-scale tissue culture and time frame of the experiment (6-9 months). Sample availability of stored mouse tumours determined sample size for WGS. For CLL-GEL , CLL-ICGC, PCAWG and Gene4Denovo, sample size was determined by available data. |
| Data exclusions | CLL-GeL: samples were excluded from analysis on pre-established criteria of <50% tumor cellularity. Subsequently, 6 further cases were excluded as indel calling with Mutect2, Platypus and/or SvABA failed. Likewise, for mouse intestinal tumours, n=3 were excluded due to low cellularity, on the basis of median SNV MAF<10%. |
| Replication | The number of times each experiment was repeated with similar results is stated in figure legends or the Methods section. Fluctuation assays were performed with a minimum of 9 independent cultures. The mutation accumulation experiment was performed once with 2 independent KO clones and 3 independent WT clones. All attempts at replication were successful for the experiments described in the manuscript. |
| Randomization | Samples were allocated to groups on the basis of genotype of interest (e.g. RNase H2 status). No randomization was performed. |
| Blinding | The investigator performing the fluctuation assays with the HeLa reporter cells was blinded to the identity of the different clones. RNASEH2B genotypic assignment for CLL samples was done prior to and independent of the person who performed the indel variant count analyses. For other experiments the investigators were not blinded during data collection and analysis, as this is not standard practice in the field (enzyme assays, alkaline gels, immunoblotting, yeast fluctuation assays). Automated colony counting was performed for fluctuation assays to avoid observer bias, and indel counts were called programmatically; therefore analyst blinding was not necessary. |

# Reporting for specific materials, systems and methods

We require information from authors about some types of materials, experimental systems and methods used in many studies. Here, indicate whether each material, system or method listed is relevant to your study. If you are not sure if a list item applies to your research, read the appropriate section before selecting a response.

## Materials & experimental systems

| n/a | Involved in the study |
|---|---|
| ☐ | ☒ Antibodies |
| ☐ | ☒ Eukaryotic cell lines |
| ☒ | ☐ Palaeontology and archaeology |
| ☐ | ☒ Animals and other organisms |
| ☐ | ☒ Human research participants |
| ☒ | ☐ Clinical data |
| ☒ | ☐ Dual use research of concern |

## Methods

| n/a | Involved in the study |
|---|---|
| ☒ | ☐ ChIP-seq |
| ☒ | ☐ Flow cytometry |
| ☒ | ☐ MRI-based neuroimaging |

# Antibodies

| | |
|---|---|
| Antibodies used | Sheep anti-pan-RNase H2 (raised against human recombinant RNase H2, 1:1,000; not commercially available, human RNase H2 was purified in the A.P.Jackson laboratory and antibody raised as part of a custom program by Eurogentec, and affinity purified using recombinant RNase H2); mouse anti-RNASEH2A G-10 (Santa Cruz Biotechnologies sc-515475, lot #A1416, 1:1,000); rabbit anti-GAPDH (Abcam ab9485, 1:2,000, lot #GR3380498-1); horseradish peroxidase (HRP)-linked Rabbit Anti-Sheep Immunoglobulins, (Dako, P04163, lot #00047199, 1:2,000); Goat Anti-Mouse Immunoglobulins/HRP-linked Antibody (Dako, P0447, lot #20039214, 1:10,000); Anti-rabbit IgG, HRP-linked Antibody (Cell Signaling Technologies, 7074S, lot #29, 1:10,000) |
| Validation | RNase H2 and RNASEH2A antibodies were previously validated using knockout cell lines (doi.org/10.1038/s41586-018-0291-z); GAPDH antibody has been previously demonstrated by the manufacturer to yield a single band of the expected size and is stated on the manufacturer's website to be cited by 2,175 publications. |

# Eukaryotic cell lines

Policy information about cell lines

| | |
|---|---|
| Cell line source(s) | HeLa cells (originally from ATCC) were a gift from G. Stewart (University of Birmingham); hTERT-RPE1 cells (originally from ATCC) were a gift from D. Durocher (The Lunenfeld–Tanenbaum Research Institute, Toronto). |
| Authentication | Cell lines were authenticated using STR DNA profiling in the labs of origin. |
| Mycoplasma contamination | All cell lines tested negative for mycoplasma contamination. |
| Commonly misidentified lines (See ICLAC register) | No commonly misidentified cell lines were used in this study. |

# Animals and other organisms

Policy information about studies involving animals; ARRIVE guidelines recommended for reporting animal research

| | |
|---|---|
| Laboratory animals | Tissues for WGS were collected from 52-week old female Villin-Cre+ Trp53-fl/fl Rnaseh2b-fl/fl mice on a C57Bl/6J background. Mice have been described previously (doi.org/10.1053/j.gastro.2018.09.047). All mice were maintained in a specific pathogen-free facility, and the quarterly health report did not indicate the presence of pathogenic species. Mice were provided with food and water ad libitum and maintained in a 12h light–dark cycle under standard conditions (ambient temperature 20-22°C, 40-60% humidity) at Kiel University. |
| Wild animals | The study did not involve wild animals. |
| Field-collected samples | The study did not involve samples collected from the field. |
| Ethics oversight | Animal experiments were conducted with appropriate permission, in accordance with guidelines for animal care of the Christian-Albrechts-University (Kiel, Germany), in agreement with national and international laws and policies. |

Note that full information on the approval of the study protocol must also be provided in the manuscript.

# Human research participants

Policy information about studies involving human research participants

| | |
|---|---|
| Population characteristics | IGCC-CLL WGS cohort: age at diagnosis 18-87 years old; 91 male, 59 female. Samples were collected before administration of any treatment.<br><br>CLL Genomics England: Demographics available for 172/198 cases: age at diagnosis 38-87y (median 65); 123 male, 49 female. WGS for 174 patients prior to treatment; 24 patients at relapse or refractory to treatment. |

| | |
|---|---|
| | CRC Genomics England: Age at diagnosis 33-81y (median 64); 71 male, 46 female. 105 patients received radiotherapy, capecitabine, irirotecan, fluorouracil or oxaliplatin treatment prior to sampling, and 12 patients were treatment-naive. |
| Recruitment | ICGC-CLL: CLL patients diagnosed at the Hospital Clínic of Barcelona or at a collaborative hospital and sent to the Hospital Clínic of Barcelona for further evaluation. Only patients that fulfilled the diagnosis of CLL or MBL (Monoclonal B-cell lymphocytosis) were included.<br><br>CLL Genomics England: Patients were treatment-naïve and in need of treatment according to iwCLL criteria (doi.org/10.1182/blood-2017-09-806398) as part of their enrollment in ARTIC, AdMIRe, RIAltO, FLAIR studies; apart from small subsets enrolled in CLEAR (early stage disease) and CLL210 (relapse refractory).<br><br>CRC Genomics England: Patient recruitment was organised by 13 Genomic Medicine Centres (GMCs) and their affiliated hospitals across the UK. |
| Ethics oversight | ICGC-CLL: All patients gave informed consent for participation in the study following the International Cancer Genome Consortium (ICGC) guidelines and the ICGC Ethics and Policy committee. The study was approved by the Research Ethics Committee of the Hospital Clínic of Barcelona.<br><br>CLL Genomics England: All patients gave written informed consent and the study was approved under the 100,000 Genomes Project Ethics Committee, East of England and South Cambridge Research Ethics Committee and the CLL Pilot Ethics approval (MREC 09/H1306/54).<br><br>CRC Genomics England: All patients gave written informed consent and the study was approved under the 100,000 Genomes Project Ethics Committee. |

Note that full information on the approval of the study protocol must also be provided in the manuscript.
